# Facial cutaneous Rosai-Dorfman disease treated with pulsed dye laser: a case report and literature review^☆^

**DOI:** 10.1016/j.abd.2024.05.002

**Published:** 2024-11-08

**Authors:** Qin-Xiao Wang, Si-Yu Luo, Kai-Yi Zhou, Sheng Fang

**Affiliations:** Department of Dermatology, The First Affiliated Hospital of Chongqing Medical University, Chongqing, China

Dear Editor,

Rosai-Dorfman disease (RDD) is a benign proliferative disease of histiocytes with heterogeneous presentation. The exclusively cutaneous Rosai-Dorfman disease (CRDD), is considered rare, accounting for about 3% of RDD cases.^1^ To date, there are no standardized treatment guidelines for CRDD. We present a case of facial CRDD successfully treated with a pulsed dye laser and summarize the characteristics of the therapy of facial CRDD with laser and other light therapies.

A 27-year-old woman presented with a 1-year history of painless erythematous papules of the mandible without lymphadenopathy or other systemic disease. Papules gradually increase in size and number and become plaques with no obvious symptoms. Physical examination revealed clusters of red papules coalescing into plaques dotted with small papulovesicles and pustules. The results of a general physical examination were normal. Blood routine, urine routine, liver enzymes, renal function, syphilis treponemal antibody tests (TPPA and TRUST), and human immunodeficiency virus test (ELISA) were normal. The patient's initial diagnosis was acne and treated with oral minocycline. A skin biopsy was performed due to the limited efficacy and showed a prominent histiocytic infiltrate in a background of inflammatory cells including the large number of lymphocytes and plasmacytes. There were also some large histiocytes with engulfed intact lymphocytes, termed emperipolesis (Fig. 1A‒1B). Immunohistochemistry demonstrated that the histiocytes were CD68 and S-100 positives, and CD1a negative (Fig. 1C‒1D). The diagnosis of CRDD was established. Given the patient’s aesthetic preferences, pulsed dye laser therapy was chosen. After the initial treatment, there was a reduction in the size of the lesions and a decrease in redness, and the patient continued to receive three sessions of pulsed dye laser treatment at one-month intervals. The lesion was flat and remarkably improved. (Fig. 2A‒2B) There were no apparent side effects and no recurrences during the 8 months of follow-up to date.

**Figure 1:**
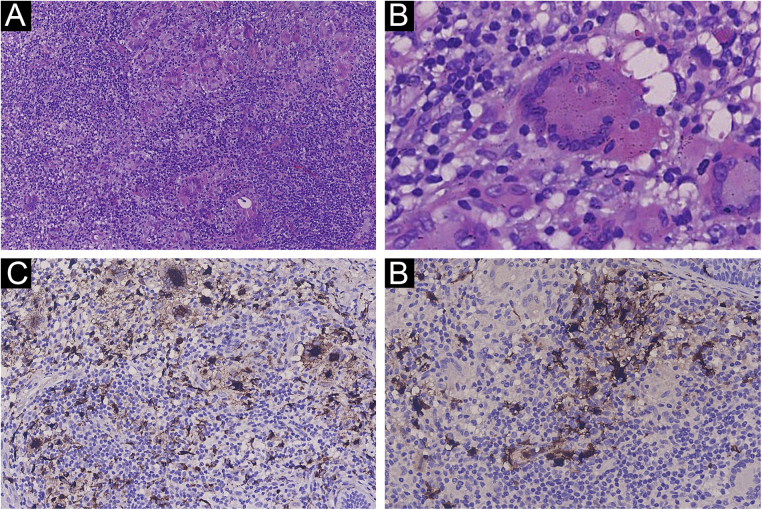
The histopathological findings and immunohistochemistry. (A) Prominent histiocytic infiltrate in a background of inflammatory cells (Hematoxylin & eosin, 100×). (B) Large histiocytes with engulfed intact lymphocyte showing emperipolesis (Hematoxylin & eosin, 400×). (C) The histiocytes showing positive for CD68. (D) The histiocytes showing positive for S100. (C, 200×; D, 200×).

**Figure 2:**
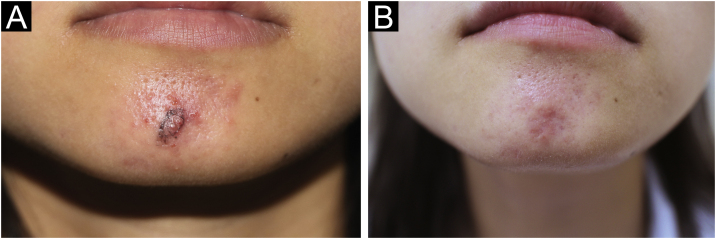
Clinical appearance of CRDD. (A) Clusters of red papules that coalesce into plaques on the mandible before treatment. (B) The lesion was flat and remarkably improved after three sessions of pulsed dye laser treatment at one-month intervals.

The treatment of CRDD is challenging due to the high recurrence rates and side effects of current therapies.^2^ Surgical excision is one of the primary treatment options, but many patients are concerned about the potential risk of postoperative complications, in particular the lesions on the face. With a lower risk of complications than conventional treatments, laser and other light therapies may be a promising treatment option for facial lesions.

A search of full-text English articles in the PubMed database identified seven cases of facial CRDD that were effectively treated with laser and other light therapies. The clinical characteristics, treatment, and outcome of the patients are summarized in Table 1.^2^, ^3^, ^4^, ^5^, ^6^, ^7^, ^8^ All the cases were from Asia and located on the face including 1 male and 7 females. The mean age was 46.4 years (range 27‒70 years) and the maximum duration of disease was 6 years. Most patients (6/8) had failed treatment prior to laser and light therapy, while two patients (2/8) were initially treated with laser and light therapy. A variety of laser and light therapies were used, including CO_2_ laser, ALA-PDT, pulsed dye laser, and fractional laser, based on the cases reported in the literature review. The evaluation period for efficacy assessment varied from 3 to 6 months, with an average of about 4.1 months after initial treatment. All patients (8/8) achieved excellent therapeutic results with no adverse events. There were no adverse events or relapses during an average follow-up of 6.9 months (range 3‒12 months).

**Table 1:** Review of previously reported cases of CRDD treated with laser and other light therapies.

Patient number	Age/Sex	Duration	Location/Size	Clinical manifestation	Previous therapy/Outcome	Treatment	Outcome	Follow-up time/ Recurrence
Case 1^2^	58/F	6 months	Left temporal; N.D.	Yellow-red nodules and plaques; asymptomatic	Oral Methotrexate, thalidomide, cyclosporine, topical glucocorticoid; refractory,	Fractional laser combined with ALA-PD for 8 weeks. Then pulse CO2 laser manual fractional pattern	CR; Basically cleared	3 months/no
Case 2^3^	39/M	9 months	Below the left nostril and left preauricular region; 3 cm × 3 cm, 0.5 cm × 1 cm	Enlarging erythematous plaques; asymptomatic	Oral thalidomide; No significant improvement, peripheral neuropathy and gastrointestinal side effects	5% daily application of imiquimod, 808-nm diode laser (3 sessions)	PR; Lesions remarkably improved	6 months/no
Case 3^4^	50/F	1 year	Both cheeks; 2-3 cm in diameter	Erythema, red-yellow nodules, and plaques; painless	None	ALA-PDT combined with low-dose oral corticosteroids; oral prednisolone 30 mg/d	CR; Almost disappeared	1 year/no
Case 4^5^	47/F	6 years	Right cheek; 0.3-1.0 cm in diameter	Enlarging reddish-brown plaques; N.D.	Topical pimecrolimus; Limited effect, local burning sensation	CO2 laser with 595-nm laser and ALA-PD for 16 weeks	CR; Completely removed	3 months/no
Case 5^6^	70/F	1 month	Philtrum; 1.5 cm × 1.5 cm	Enlarging solitary erythematous plaque; asymptomatic	Oral methotrexate and triamcinolone acetonide intracutaneous injection; ineffective	Pulsed dye laser with 595-nm laser every month for 3 months	PR; Lesion remarkably improved	10 months/no
Case 6^7^	40/F	6 months	Right cheek; 3.0 cm × 3.0 cm	Enlarging red plaque; painless, pruritic	None	Subtotal resection, ALA-PDT, 635-nm laser of every week for 6 weeks	CR; Almost disappeared	6 months/no
Case 7^8^	40/F	2 years	Left tempus; 3.5 cm × 4.0 cm	Enlarging red plaque; painless, pruritic	Oral tranilast and thalidomide; No obvious improvement	ALA-PDT; 635-nm laser every month for 6 months	PR; Lesion remarkably improved	N.D.
Our case	27/F	1 year	Mandible; 3.0 cm × 4.0 cm	Erythematous papules; painless	Oral minocycline; ineffective	Pulsed dye laser treatment at 1-month intervals for a total of three sessions	PR; Lesion flattened and remarkably improved	8 months/no

N.D., not described; CR, complete remission; PR, partial remission.

ALA-PDT, 5-aminolevulinic acid photodynamic therapy; CO_2_, carbon dioxide; F, female; M, male; PDT, Photodynamic therapy.

Evaluation time, since the beginning of treatment; Follow-up time, since the end of treatment.

Recent studies suggest that immunosuppressive macrophages, stimulated by macrophage colony stimulating factor (M-CSF), are the main mechanism involved in the pathogenesis.^9^ Photodynamic therapy (PDT) is a treatment for RDD that blocks macrophage antigen presentation and subsequent macrophage proliferation triggered by M-CSF.^2^, ^8^ The pulsed dye laser (PDL), traditionally used in vascular disease to target oxyhemoglobin for selective photothermolysis, has shown success in the treatment of other conditions. Some researchers suggest that it may play a potential role in immune regulation. It is conceivable that PDL may have similar therapeutic effects in regulating the immune system in RDD. In addition, its disruptive effect on blood vessels may reduce skin inflammation and abnormal histiocyte proliferation, contributing to the favorable outcomes observed in CRDD.^10^

In conclusion, we report a case of facial CRDD successfully treated with a pulsed dye laser. The study suggests that laser and other light therapies may be novel therapeutic strategies for the treatment of CRDD, but due to the limited amount of data available, further studies are needed to confirm this observation.

## Financial support

None declared.

## Authors’ contributions

Qin-Xiao Wang: Conceptualization; data curation; methodology; visualization; writing-original draft.

Si-Yu Luo: Data Curation; investigation; visualization.

Kai-Yi Zhou: Data curation; project administration.

Sheng Fang: Conceptualization; methodology; resources; supervision; writing-review & editing.

## Conflicts of interest

None declared.

## References

[1] Prada-Garcia M.D.C., Perandones-Gonzalez H., Linares-Navarro R. (2022). A case of cutaneous Rosai-Dorfman. Int J Dermatol.

[2] Wang H., Wang C., Wang X., Zhang L., Zhang M., Ge L. (2023). Fractional laser combined with 5-Aminolevulinic acid photodynamic therapy for the treatment of cutaneous Rosai-Dorfman disease: a case report. Photodiagnosis Photodyn Ther.

[3] Li M., Shi L., Luo M., Chen J., Wang B., Zhang F. (2017). Successful treatment of Rosai-Dorfman disease using in situ photoimmunotherapy. Indian J Dermatol Venereol Leprol.

[4] Zhou R., Wang T., Li E., Li L. (2024). Treatment of cutaneous Rosai-Dorfman disease with ALA-PDT combined with low-dose oral corticosteroids: a case report. Photodiagnosis Photodyn Ther.

[5] Song H., Wang T. (2022). 5-Aminolevulinic acid photodynamic therapy combined with CO(2) laser therapy for the treatment of cutaneous Rosai-Dorfman disease: a case report. Photodiagnosis Photodyn Ther.

[6] Park A.Y., Lee H.J., Hong S.A., Kim J.E. (2020). Successful treatment of cutaneous Rosai-Dorfman disease with pulsed dye laser. Dermatol Surg.

[7] Wan M., Ding A., Liu H., Ou J., Zhang J. (2020). Successful treatment of large cutaneous facial Rosai-Dorfman disease using combination of subtotal resection and ALA-PDT: a case report. Photodiagnosis Photodyn Ther.

[8] Sun L., Shi J., Su Z., Zhang M., Lu Y. (2018). Successful treatment of Rosai-Dorfman disease using ALA-PDT. Photodiagnosis Photodyn Ther.

[9] Middel P., Hemmerlein B., Fayyazi A., Kaboth U., Radzun H.J. (1999). Sinus histiocytosis with massive lymphadenopathy: evidence for its relationship to macrophages and for a cytokine-related disorder. Histopathology.

[10] Liu A., Moy R.L., Ross E.V., Hamzavi I., Ozog D.M. (2012). Pulsed dye laser and pulsed dye laser-mediated photodynamic therapy in the treatment of dermatologic disorders. Dermatol Surg.

